# American Academy of Dental Science

**Published:** 1894-12

**Authors:** 

**Affiliations:** American Academy Dental Science


### Reports of Society Meetings.

### AMERICAN ACADEMY OF DENTAL SCIENCE.

The regular monthly meeting of the American Academy of Dental Science was held at Young's Hotel, Wednesday evening, May 2, at six o'clock, President Smith in the chair.

*President Smith.*—Gentlemen, you will remember that at our last meeting Dr. Farlow read a very interesting paper on "Some Relations of Diseases of the Nose and Throat to Dentistry," and exhibited the patient from whom he was about to remove some adenoid growths. That operation has been performed, and Dr. Farlow kindly comes before us to-night to report the case, and to show the specimens.

[Dr. Farlow exhibited the growth which was removed by him, the operation requiring several sittings. A ten-per-cent. solution of cocaine was used. He also presented the instruments which were used in the operation, together with a number of instruments for other operations in the throat and nasal passages. The patient referred to was getting along very nicely.]

I know I express the unanimous sentiment of the Academy when I say, we all feel very grateful to Dr. Farlow for his trouble in presenting this growth, and reporting to us the results of his operations.

It now gives me great pleasure to be able to introduce to you, this evening, a gentleman who is known personally to many of you, and to all of you through his works. I have the honor of presenting Dr. Edward C. Kirk, of Philadelphia, lecturer on Operative Dentistry at the University of Pennsylvania and editor of the *Dental Cosmos*.

*Dr. Kirk.*—I will ask you, gentlemen, to consider the matter which I shall bring before you this evening as suggestive merely. I present it in the hope of stirring up a line of thought which may result in a discussion that shall be of practical value to the profession. The paper is one of the few that I have written, which I feel it necessary to preface with an apology. It was written under great stress for lack of time, and it is for that reason not up to the standard which it should be.

(For Dr. Kirk's paper, see page 746.)

DISCUSSION.

*Dr. Fillebrown.*—I should like very much to shake hands with the essayist on this subject; but as the distance is too great between us, I shall have to tender my congratulations by thought transfer. I am heartily in sympathy with his presentation of the subject, and especially so because it embodies thoughts which I have had in mind for a good while, and according to which the Harvard Dental School is now working. I do not ordinarily bring matters of "school" into a society where there are so many different interests, but I think to-night it would be quite pardonable to tell you something of what we are doing and something of how we do it. I believe the same spirit of progress is in all the dental colleges in this part of the country. I, for one, have for a long time believed that manual training is more essential than anything else in the curriculum of the school. The most important thing for a person who has met with an accident, or is suffering intensely with pain resulting from decayed or diseased teeth, is to meet some one who can treat his case practically, and bring about immediate relief, insure the return of health to the organ, and restore the tooth to usefulness. In such cases the ability to do it is much more important than a full knowledge of reasons why it should be done. Still, to make the well-rounded dentist able to worthily maintain a high professional standing and dignity, the intellectual faculties must first of all be well trained. The history of the movement which has been very accurately and clearly elaborated in the paper this evening extends back many years. The original method of dental teaching was exclusively the laboratory method, and this was considered the only essential feature of dental education, until the schools recognized the fact that a greater intellectual development was necessary, and saw that the scientific side must be magnified. This has been done at the expense of mechanical instruction and practice. In the dental department of Harvard University we have endeavored to elaborate both sides, and to provide for each department competent and practical instructors. Our method at present is to assign to each student the control of an operating chair every day in the year, and our school years are nine months long. This gives every opportunity for practical instruction and practical manipulation to fit for daily practice. Some three years ago we found it possible to adopt a preliminary course which has something of the manual training idea in it, and in which the student receives a thorough drill in the anatomy of the tooth, and performs operations on extracted teeth something after the plan suggested by Professor Weeks. They are obliged to meet a certain standard and do a certain amount of work before they are allowed to take a chair and operate upon a living patient. This has proved eminently successful, and eminently useful for the steady development of the student.

Another idea that we are now discussing, and I hope to see in good working order before long, is a system of manual training for the purpose of increasing the strength of the hand, and developing its mobility, thereby securing greater accuracy of movement. Before long I think students will be expected to show a certain amount of strength, accuracy, and adaptability of the hand, the left as well the right, before they shall attempt operations, even upon extracted teeth. I mentioned that subject two years ago in our Association of Dental Faculties, and some treated the idea with ridicule, thinking it to be unwise and impracticable. Nevertheless, I have the firm belief that this principle lies at the bottom of the education of good operators. I have been in consultation with Professor Fitz, of the Lawrence Scientific School, about what is required to accomplish this, and he is devising an apparatus, practice upon which will develop all the motions of the hand, and also of the arm and shoulder. With this instrument the muscles can be developed, their strength increased, their mobility promoted, thus insuring greater accuracy in every operator upon the teeth. The student having this training will be less likely to let the bur or excavator, or whatever instrument he may be using, go awry. I have the greatest faith in the application to dental education of the idea which has been so fully and nicely elaborated in our paper to-night.

*Dr. Eames.*—I am very much pleased to hear what has been said, the more so, because I am in hearty sympathy with it. I may say that for fifteen years I have been in the habit of using either hand in all operations about the mouth. Being naturally inclined to be left-handed, I found myself in the early part of my practice trying to use the left hand in many operations, and gradually saw some benefits arising from it, and from that onward made it a study, so that I now unconsciously use either hand, standing on either side of the chair. The advantages from this practice are very great.

The method of acquiring dexterity or manipulation with the hands and fingers cannot be better carried out than by operations upon the teeth themselves out of the mouth. It requires, I believe, greater strength to hold a tooth in position while operating upon it in the hand than when it is fixed in the head, and much benefit can be derived from the operations of excavating and filling extracted teeth. This does not lessen in any degree the possibilities of any mechanism that may be devised. In my studies under Professor Da Costa, of Philadelphia, in 1880, I received practical and valuable instruction from the clinical conferences which he established. Different members of the class were invited to make diagnoses of cases, thus giving an opportunity of comparing different thoughts, getting the different views, and correcting the wrong ones, and it seems to me that in this way, as in no other, can a method be established which will teach the student to make a good diagnosis of the more common troubles coming under the dentist's care, and also the experienced dentist in making the finer differentiation required in obscure dental diseases. For two years past I have been making use of this idea in connection with my classes at the dental school.

*Dr. Brackett.*—I must express my grateful appreciation of the merits of the paper. As I have listened to it there has passed in my mind a review of some of my own personal knowledge and experience in learning, and in teaching dentistry; the methods of dental instruction, and the results of dental education. I am impressed with the thought that I am becoming an old man in that my own experience as student and practitioner lacks only about a year of covering a quarter of a century. I have known something of the old-fashioned laboratory method of instruction, and I have been thinking about the attainments of dental practitioners of a quarter of a century ago in comparison with the attainments of the progressive practitioners of to-day. We may say that it is not fair to make a comparison because the world has made progress, and yet there is a sense in which a comparison may be made upon the basis of different methods of instruction. The previous generation of dentists began their work from the practical end, as it is called; they approached the treatment of an exposed pulp in the same manner as they did simple dental caries, and the sum total of their knowledge of dental caries, of alveolar abscess, and of the treatment of pyorrhœa alveolaris was derived from this practical experience. The opportunities for general education, cultivation, and comprehensive knowledge attained by the practitioners were very far inferior to those which we at present enjoy; and with the limitations under which they struggled and with the systems of instruction which were pursued, it is not an unjust thing to say that a majority of the men practising dentistry at that time were empirical practitioners. I do not think it is wrong to say that, even as late as 1870, few of the dental practitioners, generally speaking, including the teachers in our schools, recognized marked differences of condition in an exposed pulp. There was but little choice of treatments to be pursued; and it was plainly stated that the results of treatment would be markedly uncertain. There was but little recognition of the pathological differences in the exposed pulp. There was a similar lack of differentiation in various other pathological conditions coming under the care of the dentist, the practitioner getting his knowledge from the practical rather than from the theoretical side. The treatment was empirical, and not always well founded; it was not nicely adapted to differing pathological conditions, and was successful, therefore, in not nearly so many instances as if accompanied by the best intelligence. I think this is not a particularly slanderous statement of the condition of things that existed a quarter of a century ago, and I think the assignment of the reason that I have given is not altogether wrong.

To some educators of to-day it seems very desirable to begin at the other end and teach foundation principles; teach the students anatomy; teach them the facts of physiology; instruct them in chemistry and the principles of surgery; also the foundation and principles of pathology with especial reference to the oral cavity, and the variety of pathological expressions which such a tissue as the dental pulp may present; teach them such matters as the successive steps of inflammation,—its cause, its development, how it reaches its climax, how it may subside; and taking this as the type of multitudes of other pathological elements, if we wish to so call them, lay a broad foundation, such as shall enable students, when they come to take up the practical end of it, to do it intelligently, and to make the diagnosis, the finer differentiation, and the course of treatment in accord with foundation principles of which they have already gained a knowledge. As I understand it, speaking not only with reference to the paper, but to the profession generally, there is no contrariety of view to-day as to the necessity of such knowledge. If there are differences of opinion in our minds, it is only in the matter of relative prominence which shall be given to the two methods, and it is granted that the comparison of to-day with twenty-five years past in any particular is not a fair comparison. I do not think that this idea, in such a field as dental pathology, of teaching principles broadly and thoroughly, is a thing that can be by any means left out. I do most cordially agree with the paper that good laboratory methods should be pursued, and I know that when our essayist spoke of laboratory methods he meant the best kind, such as is pursued in our Institute of Technology, that deservedly has the reputation of fitting men well for the active responsibilities of life.

I have spoken thus not in any opposition to the doctrines of the paper, but as wishing to hold up a little of the other end, which I think should be maintained at the same time.

*Dr. L. D. Shepard.*—I wish to extend my thanks for the invitation to be present this evening and listen to the paper of my friend, whom I have regarded for so many years as one of the bright minds of our profession, and as one from whom the most was to be expected. I think the paper we have heard to-night is an ample justification of that opinion, and I have no hesitation in giving it my most thorough approbation and acceptance.

This is to be said of the ancient days: There were teachers and teachers. The majority of young men who thought of entering the profession were taken into the offices for the money which could be got out of them by their labor in the laboratory and about the office, without any serious intention to instruct and to prepare for future usefulness. I think you will grant that to be true. I think you must also grant it to be true that when the competent instructor, recognizing the solemnity of the trust which was put upon him by taking a student, was faithful to that trust, the instruction given was of such a character that as good operators were turned out as have ever been graduated from any of our dental schools,—men whose theory was, perhaps, poor, and whose knowledge was not broad, but who gave as good service to humanity as some of the more cultivated practitioners of later days. Those men live in my memory, and I have never been given to throwing a slur at any one of them because they did not possess a degree. They were noble men in the profession, and we all—certainly the older members—honor and respect them for the good they have done. Their instruction was entirely laboratory instruction, such as has been referred to, but of the good class.

Now, I think we can be critical here to-night without giving offence. I have had opportunities for testing the work of the colleges, and I have been convinced for a good many years that the great failing of our schools has been in this very question of technique. Men have come before the board for examination who were high up in theory, but their practical work was not such as should have been expected from a graduate of three years' study, and the serious question in this connection is, Why did they receive a diploma? There was lack of faithfulness somewhere in regard to the tests that had been applied. If you look at our technical schools,—the Institute of Technology has been referred to,—you will find that the student who does not make good progress during his first year is not allowed to continue in the course. I think that is true of all our reputable technical schools, and in some cases the student does not even get as far as the first year's examinations,—perhaps he only gets through six months, when he is dropped. One of the recent classes of the Institute of Technology, graduating about eighty, had a membership of four hundred at the start, and I think nearly one-half were dropped at the end of the first year. There is a " weeding out." Do the dental colleges do that? Have they done that in the past? Is the man who shows himself to be a bungler, not possessed of the inherent idea of mechanics, forbidden to come back at the end of the first year when the fact is discovered? Now, a thorough form of laboratory instruction would make that test, and the dental colleges must make that test. They must stand as guards at the gates of the profession through which no man, however well he may understand the theoretical ideas, will be allowed to pass if he shows want of aptness in mechanical dexterity; he should be stopped from progress and relegated to some profession or calling where his success will depend upon intellectual ability. I think this scheme of manual training will develop that, and if the colleges would adopt it, we should have a very much greater percentage of success in the work of our operators. In matters of scientific investigation and education our profession has made extraordinary progress during the past twenty-five years, and the dental colleges should have the chief credit, but I think I may safely say that there has not been a corresponding progress in manual dexterity. Dental machinery, the engine, and all other dental appliances, have, to a certain extent, superseded the necessity of manual dexterity, so that the difficulties which had to be met by the earlier dentists, before there was ever a rubber dam applied, can hardly be appreciated by the younger men who have been taught that the proper way to do certain operations is to use a certain appliance, and without that appliance they would not know how to begin to do it. I am firmly convinced that to be useful members of society a dentist must be a good worker. The dividing line between good to the patient and evil to the patient is sometimes exceedingly thin, and the dentist's ability to keep on the right side of that line consists, not so much in his knowledge, his science, as in his being able to accomplish desired results and overcome difficulties with the skilled hand. The successful result is due in a greater degree to skilled hand-work than to correct reasoning,—head-work. The good directing mind is a failure in a dentist unless there is also a skilled hand, but the skilled hand is a measurable success if the mind is not so well trained. Now, we must meet this question intelligently, and it seems to me the best way to do it is the adoption of the laboratory system for the training of the hand, the elimination bravely from the schools, no matter how large or how small their numbers, of those who have not the aptitude to make successful all-round dentists.

*Dr. Fillebrown.*—I want to be sure that I was understood in regard to my position on the matter of dental education. As I understand the idea of the paper to-night, it is mainly to call attention to the fact that the laboratory methods are not developed to any such degree as the scientific methods in the present system of dental education. I fully agree with that position. As has been said, in the early days of dental improvement the mechanical features received the larger share of attention, and the result was that many times the clever operator was lacking in his knowledge of anatomy, pathology, and like important studies. And the dental schools sought to remedy these deficiencies by enlarging upon the scientific methods, and they have gone on elaborating in this line until at the present time every school in the country is provided with able teachers in these branches, while, on the other hand, it is lamentable to see the number of students who are graduated with but a very little knowledge of practical manipulations.

In our dental school, while we have been in sympathy with this scientific movement throughout the land, and are continually adding to the scientific instruction, we are also enlarging and perfecting our laboratory methods. We are not neglecting either department, and we certainly do want to see the laboratory method more perfect in every detail. We want to see the student thoroughly familiar with the use of instruments, and exhibiting a fair degree of skill in their manipulation. Some time ago I examined an apparatus for the development and exercise of certain parts, called the "technicon." It is so arranged as to provide a separate movement for hand, finger, wrist, and arm practice. It can be graduated to measure the resistance used all the way from one to fifty pounds. It is an admirable machine, but, as it costs from fifteen to twenty-five dollars, it is rather expensive for use in a large class. Professor Fitz believes that he can devise and make some instrument that will cost but a few dollars, and will give the motions which are needed, and also furnish a dynamometer to show the result of the tests. I hope ere long the Harvard School will offer such practice to students, and insist that before they shall enter upon the practice of dentistry they shall have attained a certain degree of manipulative skill, and shall exhibit an average amount of strength in the various muscles which they will be called upon to use in their daily work. In this way I believe we shall lay a foundation upon which they can build a manipulative dentistry that will astonish some of our present reputable operators.

*Dr. Williams.*—As I understand the paper by Dr. Kirk, he urges that not only should the manual skill be trained, but that its application should be trained,—that is, it should be used with knowledge of principles and discrimination in their relations; for the greater the skill misapplied, the greater the blunder. I am very glad to have this idea that I have entertained for so many years so well presented and so ably advocated. I read a short paper at the Pan-American Medical Congress, that was held in Washington last September, in relation particularly to the knowledge of medical and surgical principles and training in their application to our practice, and illustrated by some cases the serious mistakes that might be made under the highest skill misapplied.

The point must not be lost sight of that the fundamental principles are just as important as the skill. We cannot reason that because a man is a good cabinet-maker, or a good jeweller, or a good engraver, therefore he is a good surgeon, or a good dentist; for if he knows nothing about the principles of the vital economy on which he is working he may fail, no matter how skilful he is.

What we need, then, is a more constant training in the exercise of manual skill with reference to the principles on which one is to apply this skill, or refrain from applying it. If we keep in mind both of these things, I think we will continue to advance; but if we confine ourselves to either one alone,—either all skill or all principles,—we are not progressing.

*President Smith.*—Do you care to close the subject, Dr. Kirk?

*Dr. Kirk.*—Yes, I would like to say a few words. In the first place, I wish to thank the members for the very interesting and very full discussion they have given to the subject of the paper. I fear, however, that a misapprehension exists in relation to some of the ideas set forth in the paper,—that is, that I have unduly emphasized what might be called the manual-training phase of the subject, and minimized the importance of the scientific side of dental education. If that inference has been formed, I wish to correct it. I fear that we use the terms "science" and "scientific" sometimes without thinking exactly what we mean by them; we pronounce them "trippingly on the tongue," disregarding or unmindful of their true meaning. Now, as I understand the term science, it means any classified system of knowledge held together by certain definite principles. As to the term scientific, I doubt if we can give an exact definition of it until the psychologist has thrown more light upon the problem of just what is involved in the mental processes which constitute the scientific method of thought. We are probably safe, however, in regarding it as a definite mode of reasoning with respect to classified knowledge. If, then, science is a system of knowledge, and thinking is to be done scientifically, it implies that we must have as a groundwork a knowledge of objects or phenomena about which we are to reason, and the purpose of my paper was to advocate the importance of obtaining our foundation knowledge of facts and phenomena at first hand from the objects themselves. It was not my intention to give undue prominence to the manual-training feature in dental education; the manual training is an incidental feature, and a means to an end. Manual training for the purpose of cultivating manipulative dexterity is a necessary factor in the development of an all-round dentist, but that must come as an incidental feature of the training, and is in a considerable degree proportioned to the natural aptitude of the student for his special kind of work. It is not a question of minimizing the importance of instruction on the scientific side, which Dr. Brackett has referred to as including physiology, chemistry, anatomy, pathology, etc., but rather an application of the laboratory system as a method for obtaining a knowledge of the facts on which those sciences are founded. We study anatomy by the laboratory method in the dissecting-room. No amount of lecturing could convey to our minds a comprehensive and permanent knowledge of anatomy; we must acquire our knowledge from the cadaver. One of the best manual-training teachers in this country has said, "Know first the thing, and then reason about it." That is the order of relationship, the method of study which I have endeavored to bring before you, and which I hope we shall be able to promote. It is not that I would emphasize the laboratory method solely as a means of acquring manual dexterity and skill, but rather as a means for enabling the student to acquire at first hand and for himself a knowledge of facts from which he can reason scientifically, and therefore correctly, thereafter. If the student does not have that kind of foundation upon which to build, his only alternative is to take his knowledge second-hand.

I might lecture for hours upon the physical properties and characteristics of an object before a class of students, but if they had never seen the thing about which I was talking I could give them only a vague and evanescent idea of it. No amount of explanation will convey to a person who has never seen an orange, for example, an exact and permanent idea of what it is; but if I pass him an orange and let him examine it, he will, in a very short time, if he observe carefully, have a grip on the peculiarities and qualities of an orange such as nothing else could give him, and he will not be likely to forget it. It is on this account, therefore, that the student should be made acquainted with the objects with which he has to deal and the phenomena which they present, their essential characteristics and relationships, and then be taught to reason correctly about them. That is the basis of the scientific method. The difficulty with the theoretical phase of instruction, which has been referred to to-night, and which has so largely been presented to the dental student heretofore, is that too often he has been taught very little of true science, and, in its stead, has been given a pseudo-science by a method of instruction founded largely upon hypotheses. What we need is a truly scientific method of instruction based upon a practical knowledge of objects and phenomena presented to the mind of the student through the medium of his own perceptive faculties.

*Dr. Williams.*—I am reminded of what the elder Agassiz used to say to us: "These, gentlemen, are mere facts; facts are stupid things, except so far as they go to elucidate a rule or prove a principle."

The facts that Dr. Kirk alludes to are necessary to the practical study of the principles which are so important, which, I suppose, he fully agrees to.

*President Smith.*—We have with us this evening a very distinguished gentleman, who has been recently elected an honorary member of this society,—Professor F. W. Putnam, Peabody professor of Archæology and Ethnology at Harvard University, and we should be very much pleased to have a few words from him.

*Professor F. W. Putnam.*—Gentlemen, I am glad to be with you. Our subjects have many points in common,—I have had a great deal to do with the teeth of the people of the past; you have a great deal to do with the teeth of the people of to-day. I have studied with much interest the differences and peculiarities in the teeth of different races, and have noted, among other things, that in some races the molars are very much smaller than in others; that in some races the third molar is not extruded from the jaw until a very much later time than in others; that in some peoples the wisdom-tooth is not always developed; and that from the anthropologist's stand-point it looks as if this wisdom-tooth was a useless and rudimentary tooth, which is gradually disappearing. . . . It is interesting in this connection to note the fact that among the Hindoos, the descendants of one of the oldest stocks of our own great branch, the wisdom-tooth does not occur in probably more than one-half of the adult population. When you meet with me in Cambridge I can show you a number of specimens among our three thousand crania which will be of interest to you, and will give us an opportunity of discussing prehistoric conditions from the skulls themselves.

I have been very much interested in the talk and paper of this evening from the stand-point of a teacher in other departments, and I agree fully with what has been said of the necessity of thorough laboratory instruction; and I heartily approve of what Dr. Shepard has said in relation to carefully observing your student during the first year; and should the student prove mentally and physically unqualified for his chosen life-work, I consider it the duty of the instructor to advise his dropping it and taking up some other branch. . . . For instance, it seems to me that some of the qualifications necessary in a student of dentistry are quickness of perception and firmness, quickness, and delicacy of touch. In the Anthropological Building of the World's Columbian Exposition we had several rooms devoted to the study of the human race, and one of the rooms was the Laboratory of Psychology, where were all the instruments for testing the sight, hearing, touch, smell, taste, memory, perception, etc., and thus judging to a certain extent by a comparison with the average of the natural mental abilities of the subject. . . . Would it not be well to subject a student of dentistry to these tests before allowing him to take up the profession? Certainly a very dull man would not make a good dentist,—that is, if I am to judge by the shining lights that I have met in that profession. He should be intelligent enough to recognize pathological conditions; he should be gentle but quick in his movements; he should possess great power in his hands, but should be delicate and accurate in his touch. . . .

In regard to laboratory instruction, it is practically what is followed in my department. Heretofore I have had students who had only graduated from other courses, but for the next year a course is offered to undergraduates. The graduate course is three years. The first thing I do with a student is to place before him a lot of stones,—some of them whole, some crushed with an iron hammer, some broken by striking one stone against another, and others carefully chipped. The student is obliged to study these specimens until he is able to describe the differences which he notices, and to tell how the peculiar formations and structures were brought about. Then I have him practise breaking the stones in various ways, and try to form similar objects to those he has been studying. In this way, while developing his reasoning and descriptive powers, he is gradually training his powers of manipulation. In the same way the student is required to study fabrics; to find out with the aid of his lens the minute characters of each specimen, to tell what it is composed of, and to describe the manner in which it is put together. For instance, one is made of reed, another is made of fibre, and another of twisted fire. After working this out in a satisfactory manner, he is given a lot of threads and required to imitate the methods of manufacture of the fabrics before him. . . . By this method he receives a thorough laboratory training as a basis for his future studies. He has, as Dr. Kirk so well described it, obtained his knowledge "first hand from the objects themselves," and putting that and his theoretical knowledge together, he will be able to understand the science as a whole. I will say still further that in my department I advise students not to refer to books in order to find out what some one else has said upon the subject until they have made a thorough study of the facts. In the graduate department the student is expected to devote himself to a general study of objects for his first two years. In the third year he takes up some special research, and is then expected to add to his observations a knowledge of all that has been written on the subject. If you give a student a book to start with, he is always depending upon authority. He never draws his own conclusions, and his knowledge of a subject is limited to what Mr. A, Mr. B, or Mr. C has written about it.

William H. Potter, D.M.D.,
*Editor American Academy Dental Science.*

